# {4,4′,6,6′-Tetra­chloro-2,2′-[2,2-dimethyl­propane-1,3-diylbis(nitrilo­methanylyl­idene)]}nickel(II)

**DOI:** 10.1107/S1600536812002085

**Published:** 2012-01-21

**Authors:** Hadi Kargar, Reza Kia, Saeideh Abbasian, Muhammad Nawaz Tahir

**Affiliations:** a Department of Chemistry, Payame Noor University, PO BOX 19395-3697 Tehran, I. R. of IRAN; b X-ray Crystallography Lab., Plasma Physics Research Center, Science and Research Branch, Islamic Azad University, Tehran, Iran; c Department of Chemistry, Science and Research Branch, Islamic Azad University, Tehran, Iran; d Department of Physics, University of Sargodha, Punjab, Pakistan

## Abstract

In the title compound, [Ni(C_19_H_16_Cl_4_N_2_O_2_)], the Ni^II^ ion is in a distorted square-planar environment coordinated by two N atoms and two O atoms of the tetra­dentate ligand. The dihedral angle between the benzene rings is 24.8 (2)°. In the crystal, mol­ecules are linked into chains along the *b* axis by weak C—H⋯O and C—H⋯Cl inter­actions. An inter­molecular Cl⋯Cl [3.4564 (19) Å] inter­action is present which is shorter than the sum of the van der Waals radii of Cl atoms (3.50 Å).

## Related literature

For applications of Schiff bases in coordination chemistry, see: Granovski *et al.* (1993 ▶); Blower *et al.* (1998 ▶). For related structures see: Ghaemi *et al.* (2011 ▶); Kargar *et al.* (2011 ▶, 2012 ▶). For standard bond lengths, see: Allen *et al.* (1987 ▶).
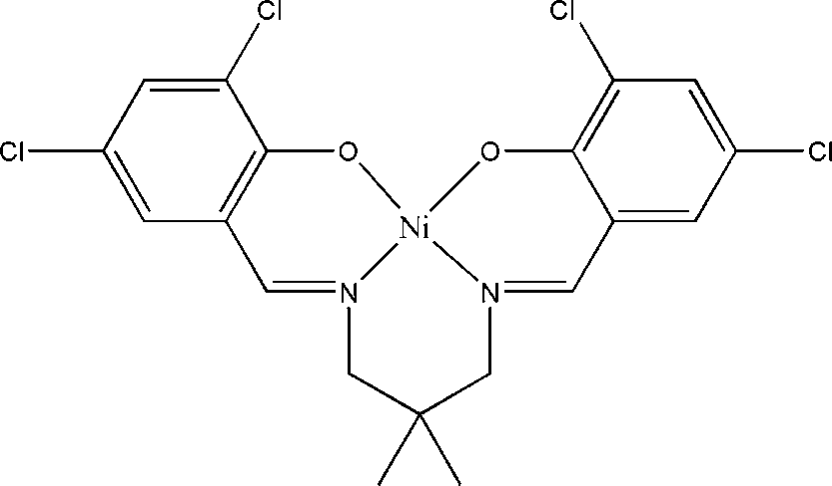


## Experimental

### 

#### Crystal data

[Ni(C_19_H_16_Cl_4_N_2_O_2_)]
*M* *_r_* = 504.85
Monoclinic, 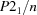

*a* = 12.4019 (8) Å
*b* = 8.1883 (6) Å
*c* = 20.3945 (13) Å
β = 96.680 (3)°
*V* = 2057.0 (2) Å^3^
*Z* = 4
Mo *K*α radiation
μ = 1.48 mm^−1^
*T* = 291 K
0.25 × 0.18 × 0.09 mm

#### Data collection

Bruker SMART APEXII CCD area-detector diffractometer
Absorption correction: multi-scan (*SADABS*; Bruker, 2005 ▶) *T*_min_ = 0.694, *T*_max_ = 0.871
17449 measured reflections
4874 independent reflections
2682 reflections with *I* > 2σ(*I*)
*R*_int_ = 0.090

#### Refinement

*R*[*F* ^2^ > 2σ(*F* ^2^)] = 0.065
*wR*(*F* ^2^) = 0.127
*S* = 1.05
4874 reflections
255 parameters
H-atom parameters constrained
Δρ_max_ = 0.49 e Å^−3^
Δρ_min_ = −0.41 e Å^−3^

### 

Data collection: *APEX2* (Bruker, 2005 ▶); cell refinement: *SAINT* (Bruker, 2005 ▶); data reduction: *SAINT*; program(s) used to solve structure: *SHELXTL* (Sheldrick, 2008 ▶); program(s) used to refine structure: *SHELXTL*; molecular graphics: *SHELXTL*; software used to prepare material for publication: *SHELXTL* and *PLATON* (Spek, 2009 ▶).

## Supplementary Material

Crystal structure: contains datablock(s) global, I. DOI: 10.1107/S1600536812002085/lh5404sup1.cif

Structure factors: contains datablock(s) I. DOI: 10.1107/S1600536812002085/lh5404Isup2.hkl

Additional supplementary materials:  crystallographic information; 3D view; checkCIF report

## Figures and Tables

**Table 1:** Hydrogen-bond geometry (Å, °)

*D*—H⋯*A*	*D*—H	H⋯*A*	*D*⋯*A*	*D*—H⋯*A*
C8—H8*A*⋯O1^i^	0.97	2.52	3.269 (5)	134
C12—H12*A*⋯Cl4^ii^	0.97	2.80	3.578 (5)	138

Symmetry codes: (i) 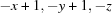; (ii) 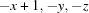.

**Fig. 1.**
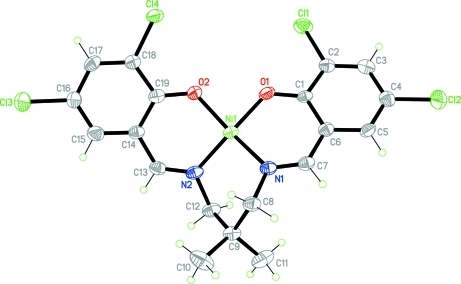
The molecular structure of the title compound, showing 40% probability displacement ellipsoids.

**Fig. 2.**
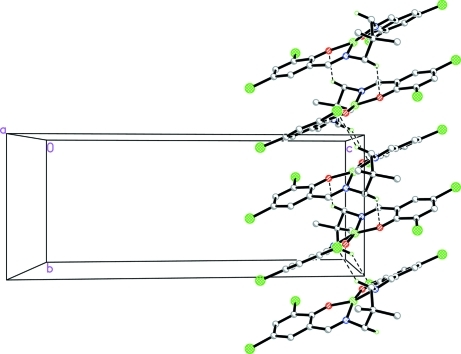
Part of the packing of the title compound showing molecules linked through intermolecular C—H···O and C—H···Cl intearctions (dashed lines). Only the H atoms involved in the interactions are shown.

**Fig. 3.**
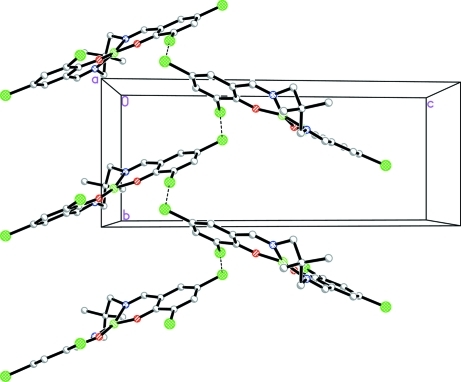
Part of the packing of the title compound viewed along the a -axis, showing molecules linked along the b-axis by intermolecular Cl···Cl intearctions(dashed lines).

## supplementary crystallographic information

### Comment

Schiff base complexes are one of the most important stereochemical models in transition metal coordination chemistry, with ease of preparation and structural variation (Granovski *et al.,* 1993; Blower *et al.,* (1998). In continuation of our work on the crystal structure of Schiff base metal complexes (Kargar *et al.,* 2012; Kargar *et al.,* 2011; Ghaemi, *et al.,* (2011), we have determined the X-ray structure of the title compound.

The asymmetric unit of the title compound, Fig. 1, comprises a Schiff base complex. The bond lengths (Allen *et al.,* 1987) and angles are within the normal ranges and are comparable to the related structure (Kargar *et al.,* 2012; Kargar *et al.,* 2011; Ghaemi, *et al.,* (2011).

The geometry around the Ni^II^ ion is a distorted square-plane which is supported by the N_2_O_2_ donor atoms of the coordinated Schiff base ligand. The dihedral angle between the substituted benzene rings is 24.8 (2)°.

In the crystal, molecules are linked along the *b*-axis, forming one-dimensional extended chains via intermolecular C—H···O and C—H···Cl interactions (Table 1, Fig. 2). In addition, Cl1···Cl2^iii^ [3.4564 (19)Å; (iii) 1/2 - *x*, -1/2 + *y*, -1/2 - *z*] interactions are present in the crystal structure which are shorter than the sum of the van der Waals radii of Cl atoms [3.50Å]. These also link neighboring molecules along the *b*-axis (Fig. 3).

### Experimental

The title compound was synthesized by adding 3,5-dichloro-salicylaldehyde-2,2-dimethyl-1, 3-propanediamine (2 mmol) to a solution of NiCl_2_. 6H_2_O (2.1 mmol) in ethanol (30 ml). The mixture was refluxed with stirring for half an hour. The resultant solution was filtered. Dark-green single crystals of the title compound suitable for *X*-ray structure determination were recrystallized from ethanol by slow evaporation of the solvents at room temperature over several days.

### Refinement

The H-atoms were included in calculated positions and treated as riding atoms: C—H = 0.93, 0.96 and 0.97 Å for CH, CH_3_ and CH_2_ H-atoms, respectively, with U_iso_ (H) = k x U_eq_(C), where k = 1.5 for CH_3_ H-atoms, and k = 1.2 for all other H-atoms.

### Crystal data

**Table:**

[Ni(C_19_H_16_Cl_4_N_2_O_2_)]	*F*(000) = 1024
*M**_r_* = 504.85	*D*_x_ = 1.630 Mg m^−^^3^
Monoclinic, *P*2_1_/*n*	Mo *K*α radiation, λ = 0.71073 Å
Hall symbol: -P 2yn	Cell parameters from 2540 reflections
*a* = 12.4019 (8) Å	θ = 2.5–27.4°
*b* = 8.1883 (6) Å	µ = 1.48 mm^−^^1^
*c* = 20.3945 (13) Å	*T* = 291 K
β = 96.680 (3)°	Block, dark-red
*V* = 2057.0 (2) Å^3^	0.25 × 0.18 × 0.09 mm
*Z* = 4	

### Data collection

**Table:**

Bruker SMART APEXII CCD area-detector diffractometer	4874 independent reflections
Radiation source: fine-focus sealed tube	2682 reflections with *I* > 2σ(*I*)
graphite	*R*_int_ = 0.090
φ and ω scans	θ_max_ = 27.9°, θ_min_ = 1.8°
Absorption correction: multi-scan (*SADABS*; Bruker, 2005)	*h* = −16→15
*T*_min_ = 0.694, *T*_max_ = 0.871	*k* = −10→7
17449 measured reflections	*l* = −26→26

### Refinement

**Table:**

Refinement on *F*^2^	Primary atom site location: structure-invariant direct methods
Least-squares matrix: full	Secondary atom site location: difference Fourier map
*R*[*F*^2^ > 2σ(*F*^2^)] = 0.065	Hydrogen site location: inferred from neighbouring sites
*wR*(*F*^2^) = 0.127	H-atom parameters constrained
*S* = 1.05	*w* = 1/[σ^2^(*F*_o_^2^) + (0.0401*P*)^2^ + 1.6535*P*] where *P* = (*F*_o_^2^ + 2*F*_c_^2^)/3
4874 reflections	(Δ/σ)_max_ < 0.001
255 parameters	Δρ_max_ = 0.49 e Å^−^^3^
0 restraints	Δρ_min_ = −0.41 e Å^−^^3^

### Special details

**Table:**

Geometry. All esds (except the esd in the dihedral angle between two l.s. planes) are estimated using the full covariance matrix. The cell esds are taken into account individually in the estimation of esds in distances, angles and torsion angles; correlations between esds in cell parameters are only used when they are defined by crystal symmetry. An approximate (isotropic) treatment of cell esds is used for estimating esds involving l.s. planes.
Refinement. Refinement of F^2^ against ALL reflections. The weighted R-factor wR and goodness of fit S are based on F^2^, conventional R-factors R are based on F, with F set to zero for negative F^2^. The threshold expression of F^2^ > 2sigma(F^2^) is used only for calculating R-factors(gt) etc. and is not relevant to the choice of reflections for refinement. R-factors based on F^2^ are statistically about twice as large as those based on F, and R- factors based on ALL data will be even larger.

### Fractional atomic coordinates and isotropic or equivalent isotropic displacement parameters (Å^2^)

**Table:**

	*x*	*y*	*z*	*U*_iso_*/*U*_eq_	
C1	0.4518 (3)	0.3786 (5)	−0.1322 (2)	0.0284 (10)	
C2	0.3696 (3)	0.3864 (6)	−0.1865 (2)	0.0329 (11)	
C3	0.3866 (4)	0.4565 (6)	−0.2451 (2)	0.0396 (12)	
H3	0.3311	0.4580	−0.2799	0.047*	
C4	0.4868 (4)	0.5257 (6)	−0.2527 (2)	0.0390 (12)	
C5	0.5684 (4)	0.5219 (6)	−0.2020 (2)	0.0371 (12)	
H5	0.6350	0.5693	−0.2073	0.044*	
C6	0.5530 (3)	0.4470 (6)	−0.1417 (2)	0.0322 (11)	
C7	0.6396 (3)	0.4527 (5)	−0.0889 (2)	0.0331 (11)	
H7	0.7009	0.5123	−0.0961	0.040*	
C8	0.7307 (3)	0.4234 (6)	0.0187 (2)	0.0371 (12)	
H8A	0.7009	0.4640	0.0574	0.044*	
H8B	0.7742	0.5098	0.0025	0.044*	
C9	0.8032 (3)	0.2782 (5)	0.0382 (2)	0.0338 (11)	
C10	0.8628 (5)	0.3121 (7)	0.1068 (3)	0.0632 (17)	
H10A	0.8112	0.3181	0.1383	0.095*	
H10B	0.9011	0.4138	0.1062	0.095*	
H10C	0.9135	0.2256	0.1189	0.095*	
C11	0.8856 (4)	0.2535 (7)	−0.0109 (3)	0.0626 (16)	
H11A	0.9338	0.3455	−0.0091	0.094*	
H11B	0.8481	0.2436	−0.0546	0.094*	
H11C	0.9266	0.1559	0.0002	0.094*	
C12	0.7341 (3)	0.1245 (6)	0.0386 (2)	0.0382 (12)	
H12A	0.7265	0.0769	−0.0052	0.046*	
H12B	0.7717	0.0459	0.0687	0.046*	
C13	0.6000 (4)	0.0806 (6)	0.1105 (2)	0.0356 (11)	
H13	0.6551	0.0239	0.1356	0.043*	
C14	0.4951 (3)	0.0785 (6)	0.1330 (2)	0.0320 (11)	
C15	0.4848 (4)	0.0125 (6)	0.1952 (2)	0.0442 (13)	
H15	0.5461	−0.0254	0.2213	0.053*	
C16	0.3867 (4)	0.0033 (6)	0.2179 (2)	0.0447 (13)	
C17	0.2938 (4)	0.0556 (6)	0.1791 (2)	0.0409 (12)	
H17	0.2265	0.0493	0.1949	0.049*	
C18	0.3019 (3)	0.1168 (6)	0.1174 (2)	0.0325 (11)	
C19	0.4025 (3)	0.1323 (5)	0.0910 (2)	0.0310 (11)	
Cl1	0.24344 (10)	0.30295 (16)	−0.17810 (7)	0.0511 (4)	
Cl2	0.50613 (11)	0.6188 (2)	−0.32739 (6)	0.0619 (4)	
Cl3	0.37569 (12)	−0.0781 (2)	0.29595 (7)	0.0770 (5)	
Cl4	0.18413 (9)	0.18304 (15)	0.07001 (6)	0.0390 (3)	
N1	0.6402 (3)	0.3834 (5)	−0.03285 (17)	0.0314 (9)	
N2	0.6250 (3)	0.1539 (5)	0.05842 (18)	0.0330 (9)	
Ni1	0.52748 (4)	0.26003 (7)	−0.00440 (3)	0.02984 (17)	
O1	0.4305 (2)	0.3113 (4)	−0.07708 (14)	0.0357 (8)	
O2	0.4051 (2)	0.1881 (4)	0.03171 (14)	0.0342 (7)	

### Atomic displacement parameters (Å^2^)

**Table:**

	*U*^11^	*U*^22^	*U*^33^	*U*^12^	*U*^13^	*U*^23^
C1	0.022 (2)	0.025 (3)	0.039 (2)	0.0036 (19)	0.0069 (19)	−0.005 (2)
C2	0.031 (2)	0.027 (3)	0.041 (3)	0.001 (2)	0.002 (2)	−0.003 (2)
C3	0.035 (3)	0.038 (3)	0.043 (3)	0.004 (2)	−0.002 (2)	−0.006 (2)
C4	0.043 (3)	0.036 (3)	0.039 (3)	0.010 (2)	0.006 (2)	0.000 (2)
C5	0.027 (2)	0.045 (3)	0.041 (3)	−0.003 (2)	0.011 (2)	0.000 (2)
C6	0.030 (2)	0.029 (3)	0.039 (3)	0.001 (2)	0.0066 (19)	−0.002 (2)
C7	0.028 (2)	0.027 (3)	0.044 (3)	−0.005 (2)	0.005 (2)	−0.004 (2)
C8	0.031 (2)	0.036 (3)	0.044 (3)	−0.008 (2)	−0.001 (2)	−0.005 (2)
C9	0.028 (2)	0.027 (3)	0.046 (3)	0.000 (2)	0.003 (2)	−0.006 (2)
C10	0.061 (4)	0.053 (4)	0.069 (4)	0.001 (3)	−0.024 (3)	−0.003 (3)
C11	0.041 (3)	0.061 (4)	0.091 (4)	−0.005 (3)	0.025 (3)	−0.001 (3)
C12	0.031 (3)	0.031 (3)	0.054 (3)	0.005 (2)	0.009 (2)	−0.008 (2)
C13	0.030 (2)	0.030 (3)	0.046 (3)	0.001 (2)	−0.001 (2)	0.003 (2)
C14	0.027 (2)	0.028 (3)	0.041 (3)	0.001 (2)	0.004 (2)	−0.001 (2)
C15	0.035 (3)	0.043 (4)	0.053 (3)	−0.001 (2)	0.001 (2)	0.014 (3)
C16	0.041 (3)	0.052 (4)	0.042 (3)	0.000 (2)	0.007 (2)	0.013 (2)
C17	0.034 (3)	0.040 (3)	0.051 (3)	−0.008 (2)	0.015 (2)	0.003 (2)
C18	0.032 (2)	0.026 (3)	0.040 (3)	0.001 (2)	0.006 (2)	−0.001 (2)
C19	0.028 (2)	0.021 (3)	0.044 (3)	−0.0025 (19)	0.005 (2)	−0.005 (2)
Cl1	0.0323 (6)	0.0514 (9)	0.0667 (8)	−0.0087 (6)	−0.0056 (6)	0.0055 (7)
Cl2	0.0578 (9)	0.0838 (12)	0.0461 (8)	0.0107 (8)	0.0139 (6)	0.0175 (7)
Cl3	0.0539 (9)	0.1181 (15)	0.0603 (9)	0.0003 (9)	0.0124 (7)	0.0444 (9)
Cl4	0.0269 (6)	0.0416 (8)	0.0489 (7)	0.0010 (5)	0.0061 (5)	0.0017 (6)
N1	0.0261 (19)	0.029 (2)	0.039 (2)	−0.0021 (16)	0.0024 (16)	−0.0054 (18)
N2	0.0257 (19)	0.028 (2)	0.046 (2)	0.0007 (17)	0.0076 (17)	−0.0085 (19)
Ni1	0.0238 (3)	0.0288 (4)	0.0374 (3)	−0.0009 (3)	0.0056 (2)	−0.0023 (3)
O1	0.0227 (15)	0.041 (2)	0.0445 (18)	−0.0029 (14)	0.0090 (13)	0.0015 (15)
O2	0.0277 (16)	0.040 (2)	0.0354 (17)	−0.0030 (14)	0.0052 (13)	0.0016 (14)

### Geometric parameters (Å, °)

**Table:**

C1—O1	1.306 (5)	C11—H11A	0.9600
C1—C6	1.408 (6)	C11—H11B	0.9600
C1—C2	1.417 (5)	C11—H11C	0.9600
C2—C3	1.363 (6)	C12—N2	1.477 (5)
C2—Cl1	1.734 (5)	C12—H12A	0.9700
C3—C4	1.392 (6)	C12—H12B	0.9700
C3—H3	0.9300	C13—N2	1.289 (5)
C4—C5	1.360 (6)	C13—C14	1.429 (6)
C4—Cl2	1.744 (5)	C13—H13	0.9300
C5—C6	1.408 (6)	C14—C15	1.397 (6)
C5—H5	0.9300	C14—C19	1.421 (6)
C6—C7	1.430 (6)	C15—C16	1.354 (7)
C7—N1	1.276 (5)	C15—H15	0.9300
C7—H7	0.9300	C16—C17	1.387 (6)
C8—N1	1.482 (5)	C16—Cl3	1.746 (5)
C8—C9	1.516 (6)	C17—C18	1.369 (6)
C8—H8A	0.9700	C17—H17	0.9300
C8—H8B	0.9700	C18—C19	1.421 (6)
C9—C12	1.523 (6)	C18—Cl4	1.742 (4)
C9—C11	1.524 (7)	C19—O2	1.297 (5)
C9—C10	1.529 (6)	N1—Ni1	1.871 (4)
C10—H10A	0.9600	N2—Ni1	1.870 (4)
C10—H10B	0.9600	Ni1—O1	1.846 (3)
C10—H10C	0.9600	Ni1—O2	1.858 (3)
			
O1—C1—C6	124.1 (4)	H11A—C11—H11C	109.5
O1—C1—C2	119.5 (4)	H11B—C11—H11C	109.5
C6—C1—C2	116.5 (4)	N2—C12—C9	113.6 (4)
C3—C2—C1	122.3 (4)	N2—C12—H12A	108.8
C3—C2—Cl1	119.0 (3)	C9—C12—H12A	108.8
C1—C2—Cl1	118.7 (3)	N2—C12—H12B	108.8
C2—C3—C4	120.0 (4)	C9—C12—H12B	108.8
C2—C3—H3	120.0	H12A—C12—H12B	107.7
C4—C3—H3	120.0	N2—C13—C14	125.9 (4)
C5—C4—C3	120.1 (4)	N2—C13—H13	117.1
C5—C4—Cl2	120.5 (4)	C14—C13—H13	117.1
C3—C4—Cl2	119.4 (4)	C15—C14—C19	120.9 (4)
C4—C5—C6	120.6 (4)	C15—C14—C13	118.7 (4)
C4—C5—H5	119.7	C19—C14—C13	120.3 (4)
C6—C5—H5	119.7	C16—C15—C14	120.8 (4)
C5—C6—C1	120.5 (4)	C16—C15—H15	119.6
C5—C6—C7	118.5 (4)	C14—C15—H15	119.6
C1—C6—C7	120.8 (4)	C15—C16—C17	120.5 (5)
N1—C7—C6	125.9 (4)	C15—C16—Cl3	120.1 (4)
N1—C7—H7	117.1	C17—C16—Cl3	119.3 (4)
C6—C7—H7	117.1	C18—C17—C16	119.5 (4)
N1—C8—C9	113.0 (4)	C18—C17—H17	120.3
N1—C8—H8A	109.0	C16—C17—H17	120.3
C9—C8—H8A	109.0	C17—C18—C19	122.9 (4)
N1—C8—H8B	109.0	C17—C18—Cl4	118.5 (4)
C9—C8—H8B	109.0	C19—C18—Cl4	118.6 (3)
H8A—C8—H8B	107.8	O2—C19—C18	120.2 (4)
C8—C9—C12	109.4 (4)	O2—C19—C14	124.5 (4)
C8—C9—C11	110.7 (4)	C18—C19—C14	115.3 (4)
C12—C9—C11	108.3 (4)	C7—N1—C8	117.6 (4)
C8—C9—C10	107.9 (4)	C7—N1—Ni1	126.2 (3)
C12—C9—C10	110.9 (4)	C8—N1—Ni1	115.5 (3)
C11—C9—C10	109.6 (4)	C13—N2—C12	117.8 (4)
C9—C10—H10A	109.5	C13—N2—Ni1	125.6 (3)
C9—C10—H10B	109.5	C12—N2—Ni1	115.4 (3)
H10A—C10—H10B	109.5	O1—Ni1—O2	84.50 (12)
C9—C10—H10C	109.5	O1—Ni1—N2	164.78 (14)
H10A—C10—H10C	109.5	O2—Ni1—N2	94.25 (15)
H10B—C10—H10C	109.5	O1—Ni1—N1	93.97 (14)
C9—C11—H11A	109.5	O2—Ni1—N1	165.55 (14)
C9—C11—H11B	109.5	N2—Ni1—N1	90.93 (16)
H11A—C11—H11B	109.5	C1—O1—Ni1	127.5 (3)
C9—C11—H11C	109.5	C19—O2—Ni1	126.2 (3)
			
O1—C1—C2—C3	179.3 (4)	C17—C18—C19—C14	0.5 (7)
C6—C1—C2—C3	−0.2 (7)	Cl4—C18—C19—C14	178.9 (3)
O1—C1—C2—Cl1	−0.9 (6)	C15—C14—C19—O2	−176.9 (4)
C6—C1—C2—Cl1	179.6 (3)	C13—C14—C19—O2	−1.2 (7)
C1—C2—C3—C4	−1.0 (7)	C15—C14—C19—C18	1.2 (6)
Cl1—C2—C3—C4	179.3 (4)	C13—C14—C19—C18	176.8 (4)
C2—C3—C4—C5	0.7 (7)	C6—C7—N1—C8	−171.3 (4)
C2—C3—C4—Cl2	−178.6 (4)	C6—C7—N1—Ni1	−1.3 (7)
C3—C4—C5—C6	0.6 (7)	C9—C8—N1—C7	−115.1 (5)
Cl2—C4—C5—C6	179.9 (4)	C9—C8—N1—Ni1	73.8 (4)
C4—C5—C6—C1	−1.7 (7)	C14—C13—N2—C12	−172.4 (4)
C4—C5—C6—C7	−177.2 (4)	C14—C13—N2—Ni1	−5.8 (7)
O1—C1—C6—C5	−178.0 (4)	C9—C12—N2—C13	−118.9 (4)
C2—C1—C6—C5	1.5 (6)	C9—C12—N2—Ni1	73.2 (4)
O1—C1—C6—C7	−2.6 (7)	C13—N2—Ni1—O1	−92.3 (7)
C2—C1—C6—C7	176.8 (4)	C12—N2—Ni1—O1	74.5 (7)
C5—C6—C7—N1	−176.0 (4)	C13—N2—Ni1—O2	−7.7 (4)
C1—C6—C7—N1	8.6 (7)	C12—N2—Ni1—O2	159.2 (3)
N1—C8—C9—C12	−37.5 (5)	C13—N2—Ni1—N1	158.8 (4)
N1—C8—C9—C11	81.8 (5)	C12—N2—Ni1—N1	−34.4 (3)
N1—C8—C9—C10	−158.3 (4)	C7—N1—Ni1—O1	−7.7 (4)
C8—C9—C12—N2	−33.1 (5)	C8—N1—Ni1—O1	162.5 (3)
C11—C9—C12—N2	−153.9 (4)	C7—N1—Ni1—O2	−91.0 (7)
C10—C9—C12—N2	85.8 (5)	C8—N1—Ni1—O2	79.2 (6)
N2—C13—C14—C15	−171.2 (5)	C7—N1—Ni1—N2	157.9 (4)
N2—C13—C14—C19	13.0 (7)	C8—N1—Ni1—N2	−31.9 (3)
C19—C14—C15—C16	−2.2 (7)	C6—C1—O1—Ni1	−10.1 (6)
C13—C14—C15—C16	−177.9 (5)	C2—C1—O1—Ni1	170.4 (3)
C14—C15—C16—C17	1.5 (8)	O2—Ni1—O1—C1	178.9 (4)
C14—C15—C16—Cl3	−179.5 (4)	N2—Ni1—O1—C1	−95.1 (7)
C15—C16—C17—C18	0.1 (8)	N1—Ni1—O1—C1	13.3 (4)
Cl3—C16—C17—C18	−178.8 (4)	C18—C19—O2—Ni1	165.5 (3)
C16—C17—C18—C19	−1.2 (7)	C14—C19—O2—Ni1	−16.6 (6)
C16—C17—C18—Cl4	−179.5 (4)	O1—Ni1—O2—C19	−176.6 (4)
C17—C18—C19—O2	178.6 (4)	N2—Ni1—O2—C19	18.6 (4)
Cl4—C18—C19—O2	−3.0 (6)	N1—Ni1—O2—C19	−92.1 (6)

### Hydrogen-bond geometry (Å, °)

**Table:**

*D*—H···*A*	*D*—H	H···*A*	*D*···*A*	*D*—H···*A*
C8—H8A···O1^i^	0.97	2.52	3.269 (5)	134
C12—H12A···Cl4^ii^	0.97	2.80	3.578 (5)	138

Symmetry codes: (i) −*x*+1, −*y*+1, −*z*; (ii) −*x*+1, −*y*, −*z*.
